# Evaluating uncertainty-based active learning for accelerating the generalization of molecular property prediction

**DOI:** 10.1186/s13321-023-00753-5

**Published:** 2023-11-08

**Authors:** Tianzhixi Yin, Gihan Panapitiya, Elizabeth D. Coda, Emily G. Saldanha

**Affiliations:** 1 Pacific Northwest National Laboratory, 902 Battelle Blvd, Richland, WA USA; 2 The University of California, San Diego, La Jolla, CA, USA

**Keywords:** Molecular property prediction, Deep learning, Uncertainty quantification, Active learning

## Abstract

Deep learning models have proven to be a powerful tool for the prediction of molecular properties for applications including drug design and the development of energy storage materials. However, in order to learn accurate and robust structure–property mappings, these models require large amounts of data which can be a challenge to collect given the time and resource-intensive nature of experimental material characterization efforts. Additionally, such models fail to generalize to new types of molecular structures that were not included in the model training data. The acceleration of material development through uncertainty-guided experimental design has the promise to significantly reduce the data requirements and enable faster generalization to new types of materials. To evaluate the potential of such approaches for electrolyte design applications, we perform comprehensive evaluation of existing uncertainty quantification methods on the prediction of two relevant molecular properties - aqueous solubility and redox potential. We develop novel evaluation methods to probe the utility of the uncertainty estimates for both in-domain and out-of-domain data sets. Finally, we leverage selected uncertainty estimation methods for active learning to evaluate their capacity to support experimental design.

## Introduction

Quantitative structure–property relations (QSAR) models have become a central component of molecular design protocols across a wide range of application areas including drug design [1] and electrolyte development [2]. The current pipeline for electrolyte development is time- and resource-intensive due to the multiple computational and experimental requirements needed to fully characterize electrolyte performance [3–5]. The ability to rapidly and accurately screen vast libraries of potential molecular candidates for performance-relevant properties would significantly accelerate the discovery of novel materials that will be needed to meet the next-generation energy storage requirements.

While deep learning models have been proven to be a promising tool for the prediction of molecular properties from molecular structure [6–8], the practical utility of such models for the screening and discovery of molecules for targeted applications is still limited in many respects. Such practical applications typically require the ability to transfer models trained on one set of molecules with known target properties to another set of molecules which may differ in significant ways from the original training data. Due to the known tendency of such models towards overfitting [9], such predictions are often unreliable, overconfident, and poorly calibrated. This is a particular challenge for molecular property prediction efforts as relevant training data sets are often biased to certain subsets of molecular space due to the scale, variation, and data availability of this space.

In application towards high throughput virtual screening (HTVS), it is beneficial for the deep learning models to be accompanied by uncertainty quantification (UQ) capabilities to understand the reliability of the predictions. Such estimates are also crucial for the targeted acquisition of new measurements or computations to support improved model performance on previously uncertain regions of molecular space to best optimize the use of time- and effort-intensive experimental and computational resources [3–5]. Ensuring that UQ methods are informative for out-of-distribution (OOD) molecules which differ significantly from the training data is essential for the effective optimal selection of new molecules for characterization.

In the context of our study, we consider UQ to be predominantly arising from two sources: data uncertainty and model uncertainty. Data uncertainty can be due to a myriad of factors, including but not limited to data domain, data sampling bias, and data sparsity. Similarly, model uncertainty encompasses uncertainties arising from aspects such as model architecture, model parameters, and training methodology.

There are several challenges to generating accurate estimates of the uncertainty of deep learning models including the ability to accurately estimate uncertainty for both in-distribution (ID) and OOD samples. Due to the sensitivity of deep learning models to distribution shifts, uncertainty estimates for OOD molecules can suffer from inaccuracy similarly to the inaccuracies observed for molecular properties predictions in these regions of molecular structure space. Prior work [10] discusses the limitations in the data sets typically used for UQ studies, with most studies performed using standard data sets that are specific to particular use cases and few performed on real-world data. Moreover, additional work is needed to understand how UQ methods perform across different deep learning architectures [10]. In particular, there have been relatively few studies investigating UQ for graph neural network (GNN) architectures [11]. Other challenges of UQ for deep learning include method scalability to large data, adaptability to complex model architectures, and interpretability for non-experts [12].

Previous work [13] demonstrates that no single UQ method has been shown to consistently outperform others across all molecular property prediction tasks. In this study, we expand upon this existing work in several ways. First, we expand the UQ evaluation to a set of larger, more diverse data sets with target properties that are relevant for energy storage applications. Secondly, while most UQ methods are evaluated on data sampled from the same data set as the training data, we specifically target our evaluation approach to probe UQ performance on tasks relevant to *generalizing* to previously unseen molecule types. To this end, we introduce novel evaluation approaches to probe whether the UQ methods can identify OOD molecules and can successfully quantify changes in model uncertainty due to data set changes. We discover that many standard UQ approaches fail to perform well at these OOD tasks. We admit that it is indeed possible to adversarially construct data populations that pose significant challenges for extrapolation from training subsets, unless there is some a priori knowledge. While the identification of OOD molecules and the quantification of changes in model uncertainty cannot be directly correlated in a trivial manner, our approach attempts to explore these correlations within the constraints of our methodology. We believe this exploration has substantial value in progressing our understanding of model uncertainties in the context of OOD prediction. Finally, we study the relationship between UQ methods and the downstream performance of uncertainty-based active learning (AL) methods. Again, we specifically focus on the capabilities of these methods to accelerate the *generalization* of the models, which is crucial for the practical usage of these methods for material design and discovery pipelines.

In this study, we perform a comprehensive analysis of UQ and AL performance for the prediction of aqueous solubility and redox potential. These properties were selected due to their applicability for the design of aqueous organic redox flow batteries (AORFBs), which are a promising next-generation energy storage technology with the potential to address current challenges with implementing grid scale energy storage solutions [14]. However, these properties are broadly applicable across a range of electrolyte design applications.

The two main research questions we address in this study are:

1. Which uncertainty estimation approaches for deep learning-based molecular property prediction generalize across target property and modeling architecture considering both ID and OOD performance?
2. Can the data requirements and generalization capabilities of deep learning models be improved through the application of uncertainty-based active learning approaches?

Our results reveal several key limitations of current UQ and active learning approaches. We find that no single UQ approach consistently performs well across all performance metrics, indicating the selection of a UQ approach should depend on the targeted downstream application of the estimated uncertainty. We find that performance of uncertainty estimates on OOD data is a significant limitation of most UQ methods, and that density-estimation methods outperform other UQ approaches on this evaluation dimension. While the performance of UQ approaches are inconsistent across metrics, they are mostly consistent across model architectures and target properties, providing evidence of the broad applicability of the conclusions regarding which methods perform well on which metrics. We find that active learning based on a density-estimation approach leads to small improvements in the ability of models to generalize to new types of molecules more rapidly than random selection of new training data. However, the improvements are currently very modest and further development will be needed to substantially reduce the current data requirements for model training.

## Data

We focus on two molecular property prediction tasks - aqueous solubility and redox potential. The solubility data set consists of 17,149 molecules with experimental solubility measurements collected from data sets curated by Gao [15], Cui [16] and Reaxys [17], which is the largest and most diverse collection of organic solubility measurements to date [8]. The redox potential data set consists of 77,547 molecules randomly selected from the PubChem database [18] with redox potential values derived from first-principles calculation performed by density functional theory (DFT) [19]. To support structure–property prediction of these target properties we leverage a set of derived molecular descriptors to characterize the molecular structure, there are 839 features for the solubility data set and 1094 features for the redox potential data set. Details on the calculation of these descriptors as well as other details of the data set are given in [8] and [19].

## Deep learning network architectures

**Fig. 1:**
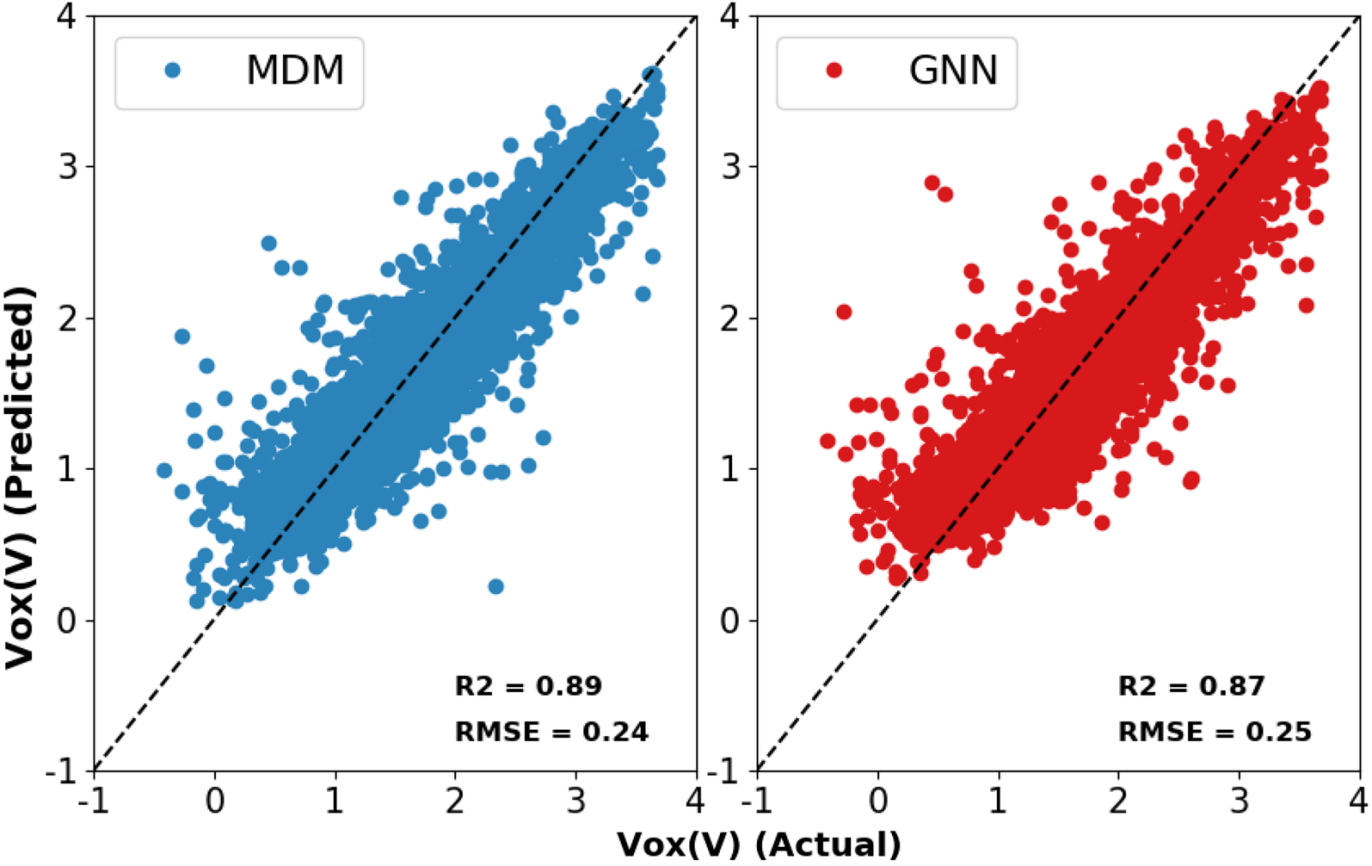
Predicted versus actual redox potential ($V_{ox}(V)$) for the MDM (left) and GNN (right) architectures including the $R^2$ and RMSE performance values

Researchers have had success in applying deep learning to QSAR models [2] in recent years. In this study, we utilize the deep learning models developed in [8], namely, the molecular descriptor model (MDM), which is a fully-connected neural network based on pre-derived molecular fingerprints, and a graph neural network (GNN) model based on molecular graphs. Details of the prediction performance of these model architectures on aqueous solubility can be found in [8], while Fig. 1 shows the predictive performance of the two architectures for redox potential.

## Uncertainty estimation approaches

A broad range of uncertainty quantification (UQ) approaches have been developed to augment the predictive capabilities of deep learning models. These approaches can be broadly categorized into four primary groups of techniques [13]. In ensemble-based methods, multiple model variants are used to generate predictions for each input, allowing the uncertainty to be quantified through the observed variance. In distance-based methods, the similarity between training and testing samples is used to quantify uncertainty. In mean-variance estimation methods, the model is tasked to predict its own uncertainty through the implementation of loss functions which supervise both the predicted mean and variance. Finally, union methods apply a second machine learning approach with more inherent uncertainty estimation capabilities in combination with the deep learning model. In this work, we explore the performance of at least one method from each category.

### Baseline

#### Gradient boosting machine

As a baseline approach, we consider a non-deep learning model which is able to generate uncertainty estimates. Specifically, we leverage a Gradient Boosting Machine (GBM) model which can provide UQ when incorporated with quantile regression [20, 21]. In the quantile regression approach, the GBM is trained to predict certain quantiles of the data in addition to the mean predictive value for the given input features. In our study, we used 10% and 90% as the lower and upper quantiles for prediction. The uncertainty is thus calculated as half of the range between the lower and upper bounds of the predictions:

1 $$\begin{aligned} \text {Uncertainty} = \frac{\text {Pred}_{90\%} - \text {Pred}_{10\%}}{2}. \end{aligned}$$

### Ensemble methods

#### Model ensemble

The ensemble approach is a straightforward approach for uncertainty estimation [22]. The idea is to train a number of structurally equivalent models and obtain the uncertainty from the variance of predictions by these models. The variance is due to the randomness in the model building process including the random weight initialization and the shuffling of training data. However, the ensemble approach is time- and resource- intensive due to the need to train multiple individual models.

#### Monte Carlo Dropout

To address the significant computational requirements of a full ensemble approach, the Monte Carlo Dropout (MCDO) approach leverages a single trained model but introduces variance when generating predictions through the application of multiple random weight dropout masks [23]. Similar to the full ensemble approach, the uncertainty is also represented by the variance in the distribution of predicted values.

### Distance methods

#### Local fingerprint and embedding density

Density-based UQ methods are motivated by the fact that molecules which are similar to the molecules in the data used to train the model should have a higher predictive confidence than those which are dissimilar to the training molecules. Therefore, we leverage a simple density-based approach to quantify the similarity of a given molecule to the training molecules. We identify the three nearest neighbors in the training data for each testing molecule and calculate the mean distance to these neighbors as the uncertainty estimation for that molecule. We explore two methods of calculating molecular similarity of the identified neighbors. First, we use cosine similarity in the molecular descriptor space, utilizing the same molecular descriptors used to train the MDM model as described in the Data Section. Secondly, we leverage the cosine similarity of pre-trained molecular embeddings generated from self-supervised pre-training of the GROVER model [24]. In contrast to other methods that we study, these methods are purely *data set* dependent rather than *model* dependent. Any model trained on the same data set will generate the same uncertainty estimate for the same molecule using these methods, which neglects any model-specific contribution to predictive uncertainty. Additionally, these methods provide only relative uncertainty estimates and do not provide calibrated uncertainty describing the range of likely errors for the molecule. Despite these limitations, as described in the results sections, we find that these methods have significant performance benefits over more expressive UQ methods under certain evaluation criteria.

### Target value modeling

#### Mean-Variance estimation

In the Mean-Variance Estimation (MVE) approach, the output of the neural network is the prediction of the mean $\mu (x)$ and variance $\sigma ^2 (x)$ of the target property [25]. The model is supervised through the application of a negative log-likelihood loss:

2 $$\begin{aligned} {\mathcal {L}}_i^{{NLL}}(x,y) = \frac{1}{2} \text {log}(2 \pi ) + \frac{1}{2} \text {log}(\sigma ^2(x)) + \frac{(y-\mu (x))^2}{2 \sigma ^2 (x)} \end{aligned}$$

The estimated variance is used as the uncertainty of the prediction.

#### Evidential deep learning

In the deep evidential regression [26], the neural network is tasked to predict the parameters of an evidential distribution of the predictive likelihood function rather than directly predicting the mean and variance of the target property. The method applies a prior distribution on the likelihood parameters $\mu (x)$ and $\sigma ^2 (x)$ and approximates the posterior distribution of these parameters using a Normal Inverse-Gamma (NIG) distribution:

3 $$\begin{aligned} p (\mu , \sigma ^2 | \gamma , \upsilon , \alpha , \beta ) = \frac{\beta ^{\alpha }\sqrt{\upsilon }}{\Gamma (\alpha ) \sqrt{2 \pi \sigma ^2}} (\frac{1}{\sigma ^2})^{(\alpha + 1)} \text {exp} \{ - \frac{2\beta + \upsilon (\gamma - \mu )^2}{2 \sigma ^2} \} \end{aligned}$$

The neural network is then tasked to learn the parameters ($\gamma , \upsilon , \alpha , \beta$) of the NIG distribution given a input datapoint. Uniquely among the methods studied in this paper, this method allows for the separation of aleatoric (${\textbf{E}} [\sigma ^2]$) and epistemic ($\text {Var} [\mu ]$) sources of uncertainty:

4 $$\begin{aligned} {\textbf{E}} [\mu ] = \gamma , {\textbf{E}} [\sigma ^2] = \frac{\beta }{\alpha - 1}, \text {Var} [\mu ] = \frac{\beta }{\upsilon (\alpha - 1)}. \end{aligned}$$

### Union approach

#### Union approach (MDM/GNN + GBM)

The union approach [27] combines the predictive capability of the deep neural network with the UQ capability of the GBM model. Features are extracted from the last representation layer of the deep learning models and used as the input variables for the GBM method. The uncertainty is then calculated as described in the GBM section above, while the output of the entire deep learning model is used as the predicted target property.

## Uncertainty evaluation metrics

We utilize four metrics to evaluate the performance of the UQ methods. These metrics are complementary to each other and probe different aspects of UQ which are salient to different downstream tasks.

### In-Distribution metrics

We first apply two metrics to probe whether the uncertainty estimates are well-calibrated and informative on new molecules drawn from the same distribution as the training data. To this end, we perform a random train-test split of the full dataset. The models and uncertainty methods are trained on the training data and UQ performance is measured on the test data. To probe the UQ performance we compare the estimated UQ values with empirically observed model errors under the criteria that high-performing UQ methods should show high correspondence between estimated UQ and actual prediction error.

#### Expected normalized calibration error

Expected Normalized Calibration Error (ENCE) measures the uncertainty calibration for regression using a histogram-based approach [28] and is designed to probe the calibration of the uncertainty estimates by comparing the estimated uncertainty with empirical error within uncertainty bins. If $\sigma _t$ is the predicted uncertainty and the samples are divided into *N* bins, $\{ B_j \}_{j=1}^N$, based on $\sigma _t$ intervals, the ENCE can be calculated as:

5 $$\begin{aligned} \textrm{ENCE} = \frac{1}{N} \sum _{j=1}^N \frac{|\textrm{RMV}(j)-\textrm{RMSE}(j)|}{\textrm{RMV}(j)}, \end{aligned}$$

**Fig. 2:**
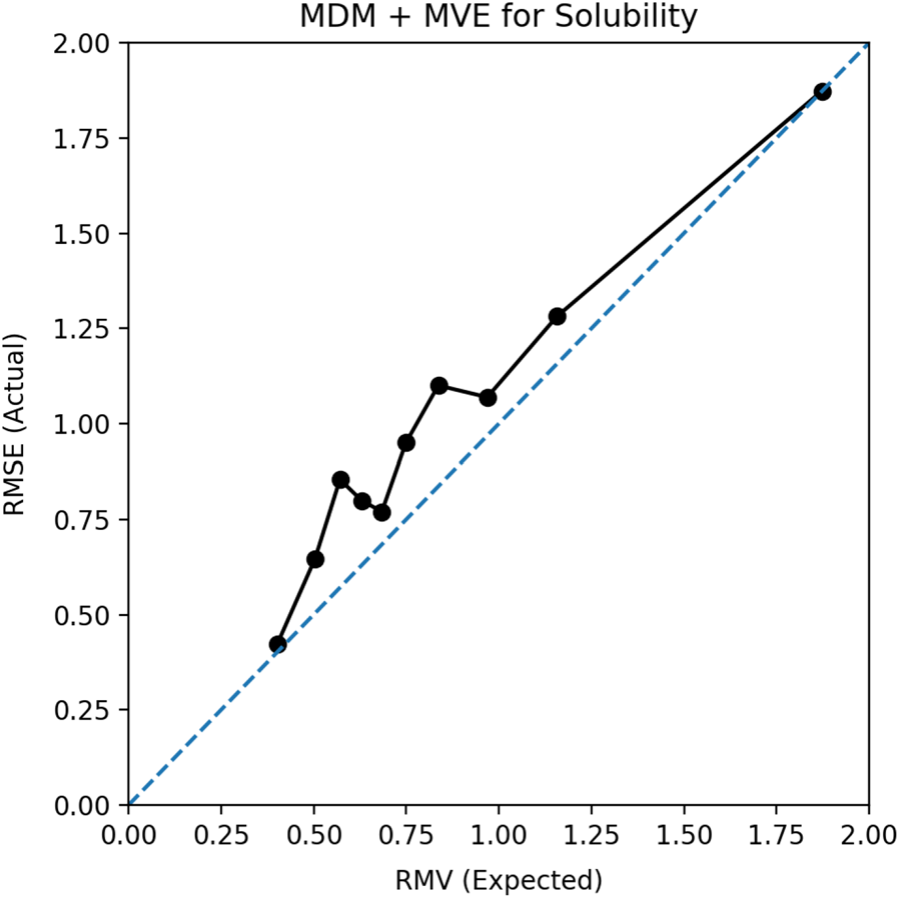
ENCE calibration example shows the correspondence between the actual model error (RMSE) and the expected error derived from the uncertainty estimate (RMV)

**Fig. 3:**
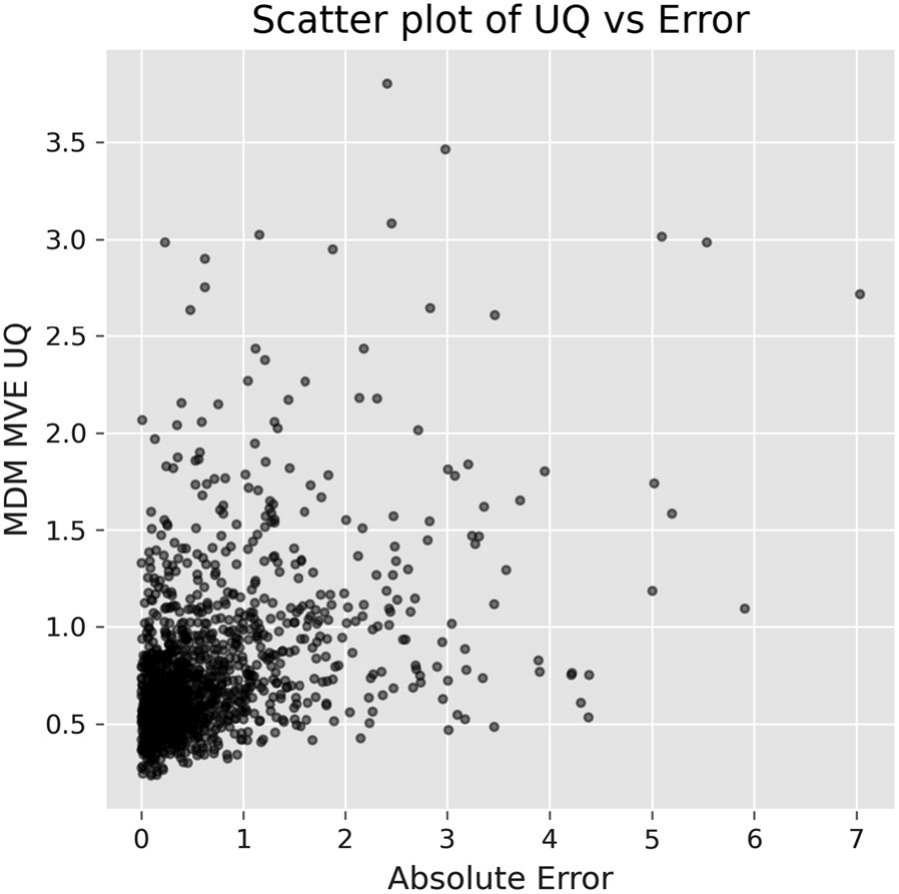
Scatter plot of the model absolute error and the UQ from MDM MVE for solubility

where RMV is the root of the mean variance:

6 $$\begin{aligned} \textrm{RMV}(j) = \sqrt{\frac{1}{|B_j|}\sum _{t \in B_j} \sigma _t^2 }, \end{aligned}$$

and RMSE is the root mean square error on the true ($y_t$) and predicted ($\hat{y_t}$) molecular properties:

7 $$\begin{aligned} {\rm{RMSE}(j)} = \sqrt{{\frac{1}{|B_j|}\sum _{t \in B_j} (y_{t}} - {{\hat{y_t})^2}}}. \end{aligned}$$

This equation expresses the average calibration error across the bins normalized by the root of the mean variance. Figure 2 shows an example of an ENCE calibration plot for the UQ provided by MDM + MVE for the solubility prediction. Fig. 3 is the scatter plot for the same method showing the relationship of the UQ and the absolute errors.

This metric provides an important performance measure of the calibration of the uncertainty estimates to probe whether the uncertainty levels are true indicators of the expected level of error when making property predictions for new molecules, which is crucial for downstream applications where the expected error values will need to be propagated to further calculations or performance estimates.

#### Error-UQ correlation

The Spearman’s rank correlation coefficient between the empirical prediction errors and the estimated uncertainty values ($\rho _{\text {error}}$) provides a probe of the relative uncertainties across the model predictions and can quantify whether the UQ correctly estimates which samples will result in higher model errors than others. For some downstream tasks, the relative level of uncertainty may be more important than the absolute calibrated value. For example, in the selection of samples for active learning, we seek to understand which regions of the molecular space are currently *most* uncertain relative to the rest. Therefore, $\rho _{\text {error}}$ provides a useful complement to ENCE to understand the UQ performance.

### Out-of-Distribution metrics

The ENCE and $\rho _{\text {error}}$ probe the performance of UQ on molecules drawn from the training distribution. When UQ is only evaluated on molecules from training distribution, it is not clear whether the uncertainty estimates will be correct when extrapolating to new data sets. We expect that molecules which are dissimilar from the training data should be estimated to have higher uncertainties compared with molecules drawn from the training data distribution. We develop two methods to evaluate the performance of the uncertainty estimation when applied to out-of-distribution molecules.

#### OOD-UQ correlation

 The $\rho _{\text {ood}}$ metric probes the ability of the UQ methods to identify OOD molecules. First, we leverage the PubChem database to sample molecules with varying structural similarity to the training data sets. We randomly sample a subset of PubChem molecules and assign each molecule a similarity score given by the maximum RDKit fingerprint similarity between the molecule and each molecule in a random subset of the training data set. Then, we downsample the PubChem molecules to obtain a final sample with similarity scores uniformly distributed over [0,1]. We calculate the Spearman’s rank correlation coefficient ($\rho _{\text {ood}}$) between the estimated uncertainty and the fingerprint similarity of these OOD molecules to the training data set. When the UQ is performing well, we expect that molecules which are more similar to the training set should have lower uncertainty. Therefore, this metric probes the relative molecule out-of-distribution detection ability of the UQ method. As with $\rho _{\text {error}}$, this metric is useful for applications such as active learning where we aim to identify undercharacterized regions of molecular space.

##### $\Delta$*Error*-$\Delta$*UQ* correlation

The first three metrics probe the absolute or relative uncertainty estimates of a given model. However, during the process of model selection, uncertainty often needs to be compared between different models to determine the relative confidence on a set of predictions. Therefore, we develop a novel evaluation approach to probe whether uncertainty estimates can robustly quantify the relative levels of uncertainty among model alternatives. First, we artificially remove molecules with certain properties from the training data to evaluate the resulting change in uncertainty when making predictions on these types of molecules. Then, we calculate the Spearman’s rank correlation coefficient ($\rho _{\Delta \text {error}}$) between change in the RMSE and the change in the estimated uncertainty for the type of molecule which was removed. This metric measures whether observed differences in uncertainty between two models (e.g. one with access to more data than the other) are meaningful in terms of the differences in the models’ errors.

**Fig. 4:**
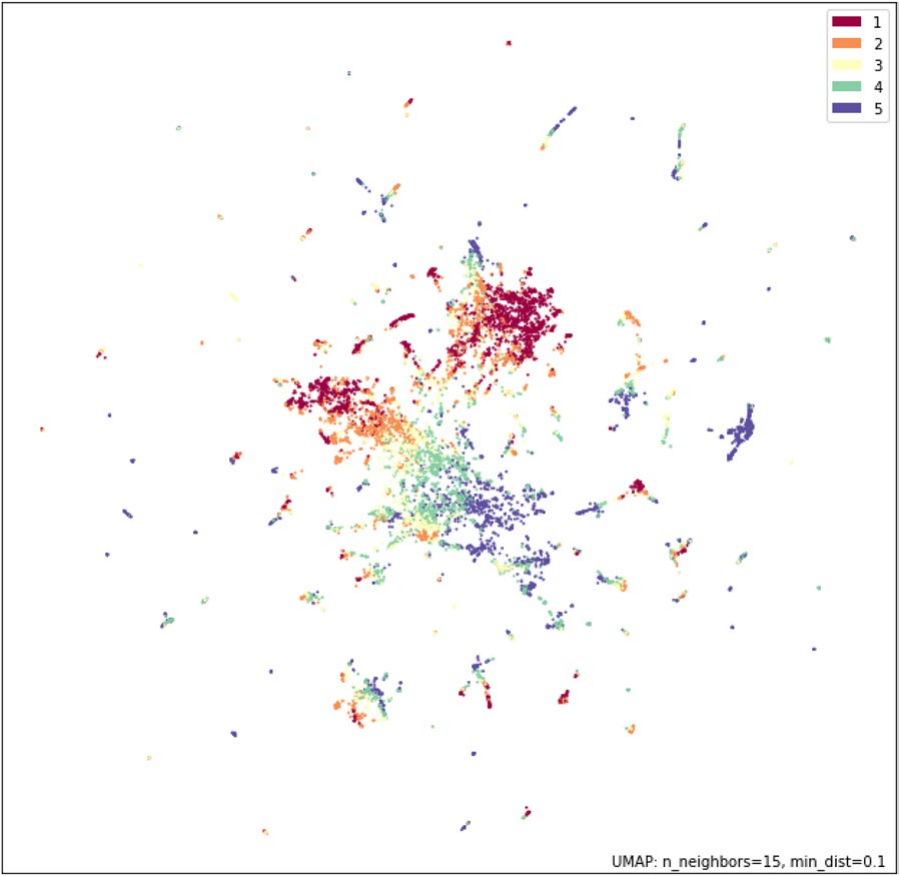
Scatterplot of the UMAP coordinates colored by the PCA bins for the top principle component

In order to create the artificial OOD data needed for this metric, we first conduct Principal Component Analysis (PCA) on the molecular descriptors. Then for each of the top three principal components (PCs), we bin the full data set into 5 equally sized bins by PC value percentiles. We then remove one bin at a time from the training and validation data sets to generate models which are trained on an artificially biased data set which does not contain molecules of a certain type. Figure 4 shows the 2D UMAP projection [29] of the training data colored by the PCA bins, which shows that the removal of each PC bin corresponds to removing specific regions of the structural space.

**Fig. 5:**
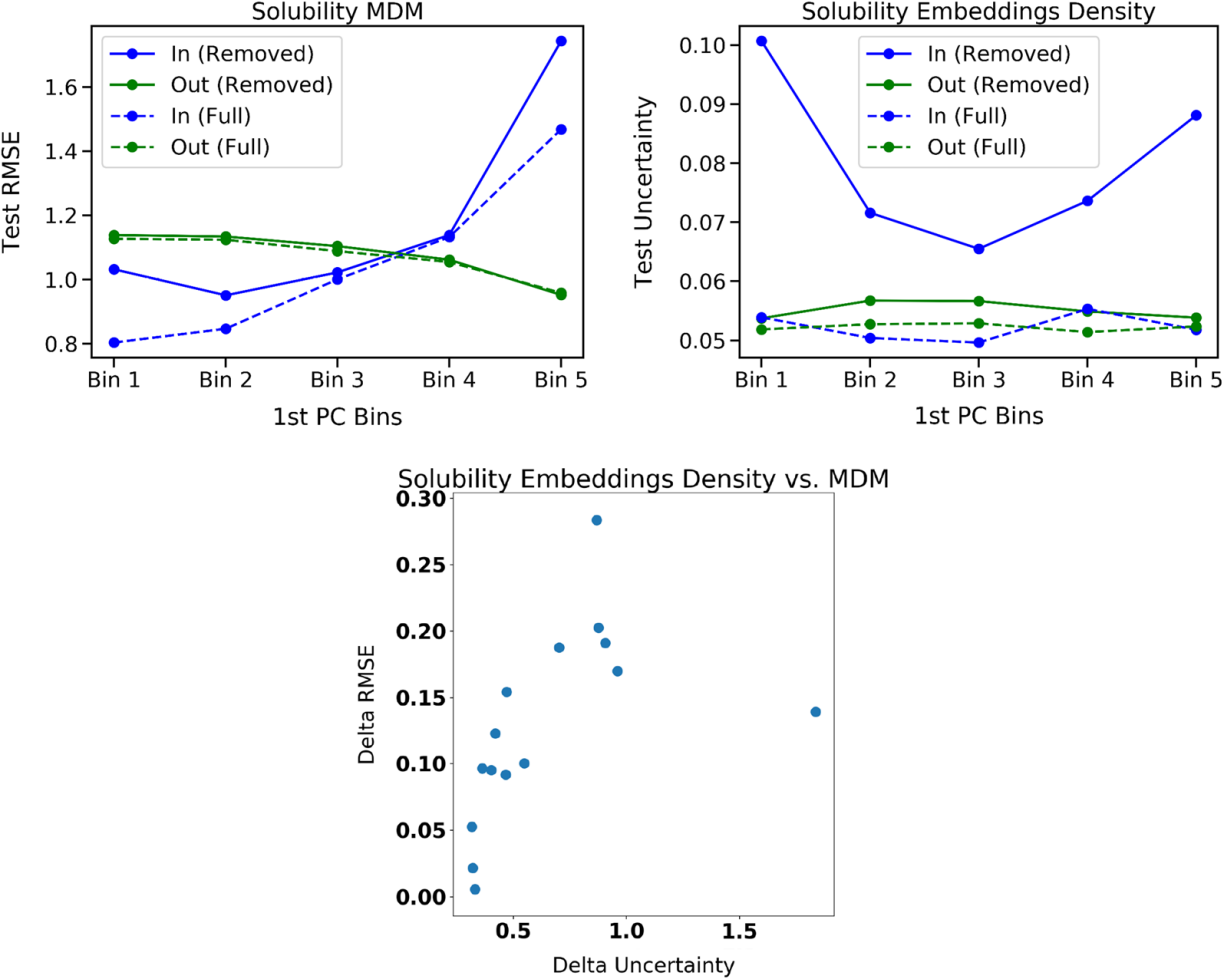
(Upper Left) Test set RMSE values increase for molecules in each PC value bin when training molecules from that bin are removed from training (blue solid line) relative to when the full training set is used (blue dashed line). RMSE values for molecule types which are not removed from the training set are not affected (green lines). (Upper Right) Uncertainty estimates generated using a density approach show that changes in uncertainty follow a similar pattern to changes in error. (Lower) The correlation of $\Delta$Error and $\Delta$UQ across all PCA components and bins is 0.818

Next, we observe how the RMSE changes for test set molecules within the removed bin before and after removing the corresponding molecules from the training and validation sets. In Fig. 5, we show an example for one model and UQ approach of how the model errors and uncertainty estimates change for molecules both within and outside the removed bin. A well performing UQ approach will show changes in uncertainty that mimic the patterns observed in changes in error. Specifically, we measure the correlation $\rho _{\Delta \text {error}}$ between the change in RMSE and the change in the uncertainty for the removed bin test molecules. Performance on this metric is important for leveraging UQ to understand improvement and changes in model performance.

## Active learning experiment

**Fig. 6:**
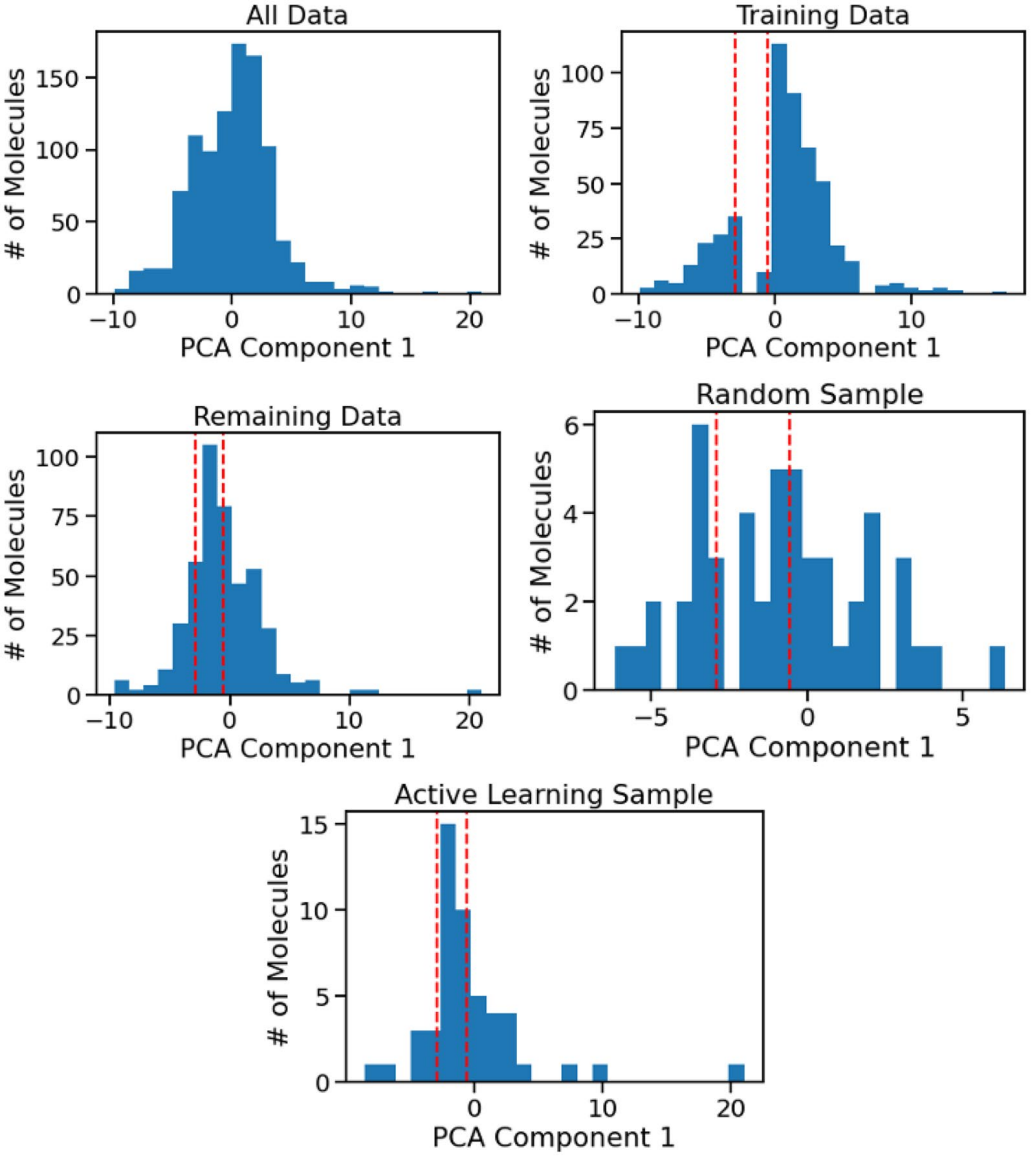
Toy example of the active learning sampling evaluation approach, illustrating the usage an artificially OOD region of molecular structure space to test the generalization performance of the sampling. The full distribution of PCA component values (Upper Left) is binned and one bin is removed from the model training data (Upper Right). The remaining data (Middle Left) is sampled to augment the initial training set, either randomly (Middle Right) or based on UQ estimates (Lower)

One end goal of developing UQ methods is the use of the estimated uncertainty for active learning that leads to more efficient guidance of molecular data collection. Active learning utilizes the information in unlabeled samples to choose the data that helps the model perform best with limited resources [30]. The motivation for the application of active learning is the time-consuming or expensive nature of obtaining new labeled training samples, which is the case for molecular research where both experimental and computational data collection is often resource, effort, and/or time intensive. Active learning has been previously used to support experimental design efforts for material characterization [3–5]. There are several different approaches which have been employed for active learning sample selection including uncertainty sampling [31, 32], query-by-committee [33, 34], expected-model-change [35], and density-weighted methods [36].

In this study, we probe the utility of several selected UQ algorithms from the previous section for active learning leveraging a query strategy which upweights more uncertain molecules. Such upweighting is linear, which means a molecule with twice as much uncertainty is twice as likely to be sampled. We aim to evaluate the ability of the UQ methods in selecting the best molecules for boosting the deep learning model prediction capability relative to the random sampling of new data points. Additionally, we focus on the potential of active learning methods to boost the *generalization* capabilities of deep learning models to adapt and generalize faster to new types of molecules with limited data availability.

We follow a similar approach to the $\rho _{\Delta \text {error}}$ calculation to understand how quickly the model can generalize to an artificially held out type of molecular structure. We first generate five bins of molecules for each of the top three principle components. To generate an initial training set for the molecules, we remove the molecules from a given bin from the data and sample the training data from the remainder of the molecules to create an artificial lack of representation for a specific group of molecules. We then train a model on this sampled training data and use the UQ methods to calculate the uncertainty for all the remaining molecules, which include those from the missing group. These uncertainty values are used to perform a weighted sampling from the remaining data. We add this active learning sample to the training data and repeat the model training procedure. This procedure is illustrated in Fig. 6. In this study, we performed one iteration of active learning. Our choice for a single iteration was intended to evaluate the immediate ability of the various active learning methods to identify OOD molecules, following the removal of a specific group from the training data.

The aim is to test the ability of the UQ methods to preferentially select OOD molecules and evaluate how the selection improves model performance relative to randomly sampling the same number of molecules. We perform evaluation on three subsets of the test set - molecules within the OOD bin, molecules in the other bins which were not removed from training, and the full test set. To measure the utility of the active learning approach we take the percentage improvement in RMSE when adding an active learning selected batch relative to a randomly selected batch.

We explore several dimensions which may affect the active learning results, including the size of the initial training set and the number of new molecules sampled using the UQ-guided active learning in the second step. For most models we perform experiments with 5%, 10%, 20%, and 40% initial training samples. For the redox potential prediction with the GBM model, which is the most time-intensive model to train, we rely on smaller samples (1%, 2.5%, 5%) to make the computation tractable. In terms of the number of molecules to sample during active learning, we experiment with 25, 50, 100, 250, 500, and 1000 additional molecules. We perform thirty repetitions with each parameter setting to increase the robustness of the measured performance results.

With four starting training percentages and six different number of added molecules, we generated a total of 24 unique combinations. Each of these combinations was subject to top three principle components binning, with each principle component further divided into five bins for OOD data simulation. This results in 15 distinct experiments for each OOD active learning scenario. Considering the combinations, there are 360 experiments in total. Furthermore, each of these OOD experiments was performed 30 times to provide a robust representation of the active learning outcomes.

**Table 1:** Solubility uncertainty estimation evaluation

UQ type	Method	Model	ENCE	$\rho _{\text {error}}$	$\rho _{\text {ood}}$	$\rho _{\Delta \text {error}}$
Baseline	GBM	GBM	0.098	$\mathbf {0.293 \pm 0.002}$	−0.191	0.386
Ensemble	MCDO	MDM	1.585	0.180	−0.219	0.404
		GNN	1.951	0.109	−0.091	0.111
	Ensemble	MDM	2.349	0.296	−0.099	−0.142
		GNN	2.830	−0.010	−0.145	0.264
Target value	Evidential	MDM	1.147	0.381	0.103	−0.404
		GNN	0.457	0.145	0.207	0.142
	MVE	MDM	$\mathbf {0.278 \pm 0.034}$	0.378	−0.037	−0.261
		GNN	$\mathbf {0.112 \pm 0.026}$	0.041	0.035	−0.314
Union	GBM	MDM	0.366	0.111	−0.371	−0.618
		GNN	0.126	0.278	−0.352	0.179
Distance	Data density (FP)	MDM	–	0.151	**0.965**	**0.857**
		GNN	–	0.143	**0.965**	**0.693**
		GBM	–	0.124	**0.965**	**0.546**
	Data density (EB)	MDM	–	0.183	0.500	0.818
		GNN	–	0.178	0.500	0.679
		GBM	–	0.142	0.500	0.539
Consensus	GBM, MCDO, MVE	MDM	0.230	0.313	–	–

The uncertainty approach with best mean performance across models for each metric is shown in bold. For the density method, FP refers to fingerprint-based similarity and EB refers to embedding-based similarity

**Table 2:** $\rho _{\Delta \text {error}}$ comparison using a different clustering method

UQ Type	Method	Model	$\rho _{\Delta \text {error}}$ (PCA Bins)	$\rho _{\Delta \text {error}}$ (Butina Clustering)
Distance	Data density (FP)	MDM	0.857	0.600
		GNN	0.693	0.400
		GBM	0.546	0.300

**Table 3:** Mean distance by fingerprint similarity between molecular groups

Group	PCA, within OOD	PCA, OOD vs. ID	Butina, within OOD	Butina, OOD vs. ID
Group 1	0.502	1.045	0.943	0.992
Group 2	0.784	0.994	0.921	0.995
Group 3	0.959	0.987	0.801	0.988
Group 4	0.924	1.039	0.774	0.961
Group 5	0.701	1.141	0.819	0.975

Larger number implies greater distance. Within OOD is the mean distance between all pairs of OOD molecules

**Table 4:** Redox potential uncertainty estimation evaluation

UQ Type	Method	Model	ENCE	$\rho _{\text {error}}$	$\rho _{\text {ood}}$	$\rho _{\Delta \text {error}}$
Baseline	GBM	GBM	0.524	$\mathbf {0.283 \pm 0.001}$	0.296	0.679
Ensemble	MCDO	MDM	1.742	0.246	0.329	0.814
		GNN	1.428	0.112	0.346	−0.461
	Ensemble	MDM	1.730	0.265	0.407	0.225
		GNN	2.417	0.028	0.405	0.229
Target value	Evidential	MDM	7.441	0.386	0.103	−0.125
		GNN	2.718	0.013	0.389	0.054
	MVE	MDM	$\mathbf {0.273 \pm 0.066}$	0.384	0.278	0.300
		GNN	$\mathbf {0.074 \pm 0.006}$	0.034	0.230	−0.118
Union	GBM	MDM	0.455	0.191	0.165	−0.296
		GNN	0.466	0.195	−0.118	−0.071
Distance	Density (EB)	MDM	–	0.199	**0.578**	**0.743**
		GNN	–	0.162	**0.578**	**0.743**
		GBM	–	0.119	**0.578**	**0.693**

The uncertainty approach with best mean performance across models for each metric is shown in bold. For the density method, EB refers to embedding-based similarity

## Uncertainty estimation results

**Fig. 7:**
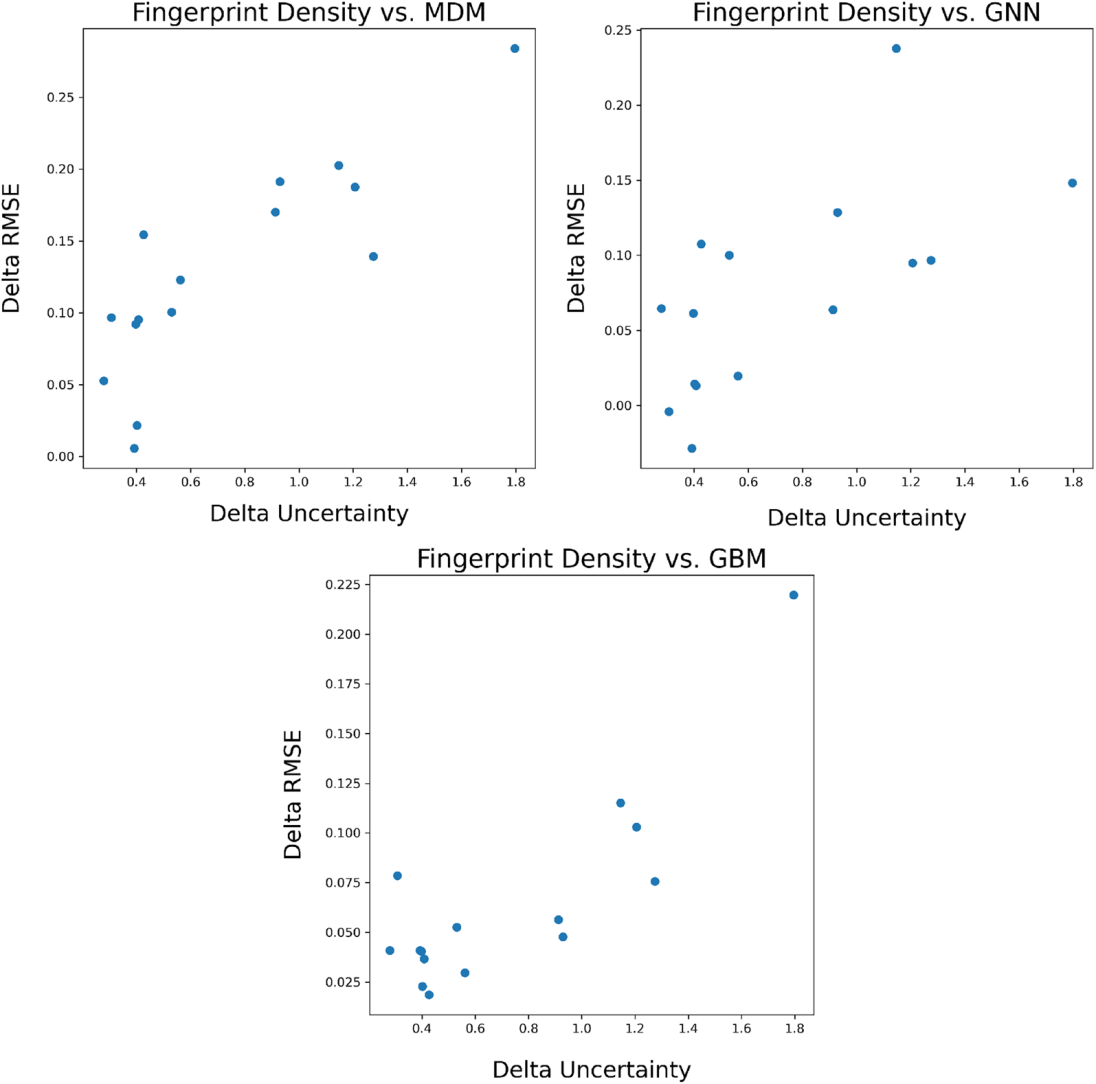
Scatter plot of the delta UQ versus delta RMSE for the fingerprint density approach on solubility

We show the full set of UQ performance results across the four metrics for solubility in Table 1 and for redox potential in Table 4. Please note that a smaller number in ENCE indicates better performance while for the other three metrics a larger number is preferable. Given the computational constraints, we focused our robustness checks on the top-performing methods for each respective metric. These results, which demonstrate the reliability of our findings, are presented with associated error bars. Regarding the density-based methodologies, they follow a deterministic process; hence, no error bars are associated with their results. We find that no single UQ approach consistently performs well across all four metrics. For example, the MVE uncertainty estimates are well calibrated according to the ENCE metric but perform poorly on generalizing to previously unseen data according to the $\rho _{\text {ood}}$ and $\rho _{\Delta \text {error}}$ metrics. In fact, we find that the majority of the UQ approaches struggle to achieve good results on these two generalization metrics. The exceptions are the density-based approaches which achieve relatively strong performance on these metrics as well as the $\rho _{\text {error}}$ metric. Figure 7 shows the scatter plots of delta UQ vs. delta RMSE for the fingerprint density approach, which corresponds to its performance for the $\rho _{\text {ood}}$ metric. However, the density-based approaches are only able to generate relative uncertainty estimates, rather than calibrated values of the expected error, meaning that ENCE is not applicable to these approaches.

**Fig. 8:**
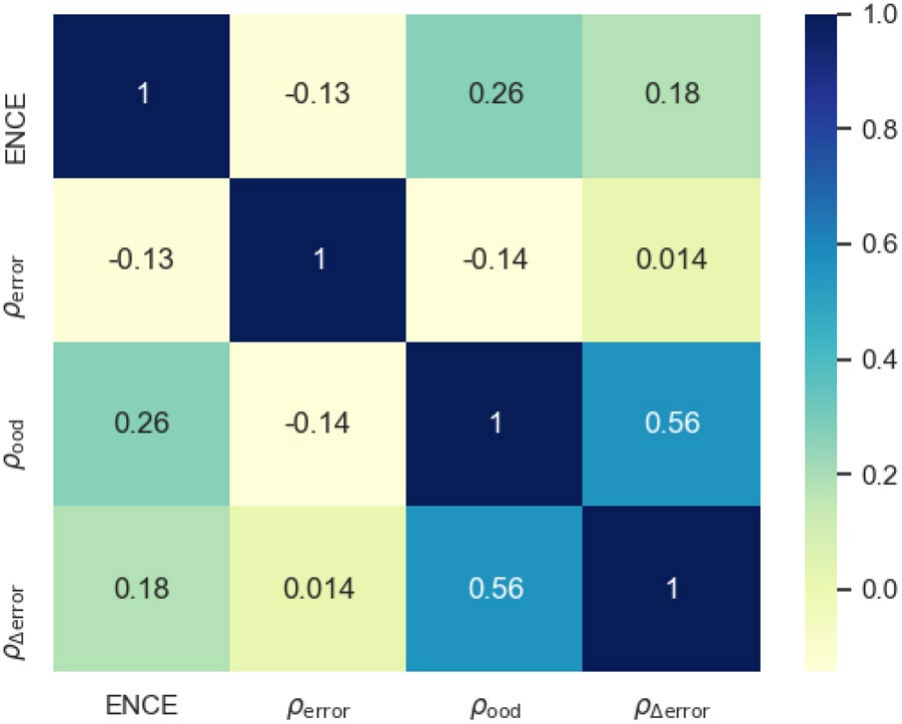
Heatmap of correlations between UQ metrics given the same UQ approach, model architecture, and target property

To illustrate this inconsistent performance, Fig. 8 shows a heatmap of correlations between UQ metrics of the same UQ approach on the same model and data set across different pairs of metrics. We find overall low levels of correlation between performance on one metric and performance on another indicating that the selection of an effective UQ approach will be strongly dependent on the ultimate downstream use case of the UQ values. For example, when the purpose is to identify the highest confidence predictions of the model then the $\rho _{\textrm{error}}$ metric may be most relevant, motivating the selection of the GBM uncertainty estimates. If the estimated uncertainty will be further propagated into an estimate of the utility of the molecule for a given application that depends on the target property, the best calibrated UQ method will be more relevant, favoring the selection of the MVE approach.

While the UQ approach performance is not consistent across different metrics, we do see some consistency of performance across different model architectures and target properties. Table 5 shows that for different modeling architectures, given the same target property, the same UQ approaches perform well relative to others for both MDM and GNN architectures for most UQ metrics. The exception is $\rho _{\text {error}}$ where UQ methods that perform well for the MDM seem to perform poorly for the GNN. For example, MVE performs strongly for the MDM model and poorly for the GNN. Table 5 also shows that for different target properties, given the same model architecture, the same UQ approaches perform well relative to others for both solubility and redox potential prediction. Overall, for these selected models and target values, UQ performance is strongly dependent on the selected metric, somewhat dependent on the underlying model architecture, and relatively unaffected by the target property.

For $\rho _{\text {ood}}$, in addition to our PCA-based grouping, we included the results from Butina clustering as a point of comparison. Butina clustering is a popular technique used in chemoinformatics to group molecules based on structural similarity [37]. By adjusting the distance threshold, we generated several sizable clusters using Butina clustering. The top five clusters comprised 5025, 748, 826, 524, and 535 molecules, respectively. We then used these clusters as our artificial OOD dataset, excluding them individually from the training, validation, and testing sets. Based on this split, we calculated the $\rho _{\text {ood}}$ for Butina clustering, and the results are presented in Table 2. Interestingly, both methods demonstrated similar relative patterns in performance.

To further compare these two grouping methods, we calculated the mean distance between OOD vs. ID groups for both the PCA and Butina methods using fingerprint similarity. These findings are shown in Table 3, where we also included the out-of-distribution mean distance as a reference point. The mean distance ranges from 0 to 2, with 2 being the most distanced. The PCA method was more effective in distinguishing between OOD and ID molecules compared to the Butina method.

We also tested a consensus method which combined the predictions and UQs from GBM (baseline), MCDO (ensemble), and MVE (target value modeling). We took the average of the predictions and UQs from these three methods. The results of this consensus method are shown in Table 1. The results show that the consensus method resulted in a better average ENCE, indicating the predictions were better calibrated. It also improved the correlation with the true errors. This indicates that the consensus method has the potential to create a better balance between the different metrics.

**Table 5:** Correlation of UQ metrics across model architectures or target properties

Metric	Dimension 1	Dimension 2	Correlation
ENCE	MDM	GNN	0.93
$\rho _{\text {error}}$	MDM	GNN	-0.66
$\rho _{\text {ood}}$	MDM	GNN	0.90
$\rho _{\Delta \text {error}}$	MDM	GNN	0.27
ENCE	Solubility	Redox	0.67
$\rho _{\text {error}}$	Solubility	Redox	0.91
$\rho _{\text {ood}}$	Solubility	Redox	0.51
$\rho _{\Delta \text {error}}$	Solubility	Redox	0.80

## Active learning results

**Table 6:** Active learning results

Target	Model	Sampling	Standard	Generalization		
			Whole	OOD Data Bin	Whole	ID Bins
Solubility	MDM	Data Density (EB)	0.14%	0.25%	0.08%	0.03%
			$\varvec{p=0.015}$	$\varvec{p=0.013}$	$p=0.146$	$p=0.326$
		Diversity (EB)	0.00%	0.13%	−0.03%	−0.07%
			$p=0.483$	$p=0.098$	$p=0.674$	$p=0.793$
		MCDO	0.13%	0.08%	0.10%	0.11%
			$\varvec{p=0.032}$	$p=0.204$	$\varvec{p=0.036}$	$\varvec{p=0.024}$
		OOD Only	–	1.93%	0.18%	−0.28%
			–	$\varvec{p<0.001}$	$\varvec{p=0.007}$	$p=0.992$
Solubility	GBM	Data density (EB)	−0.01%	0.24%	0.04%	−0.03%
			$p=0.714$	$\varvec{p<0.001}$	$p=0.084$	$p=0.868$
		GBM	0.06%	0.11%	0.05%	0.03%
			$\varvec{p=0.002}$	$\varvec{p=0.012}$	$\varvec{p=0.045}$	$p=0.203$
		OOD only	–	1.82%	0.14%	−0.31%
			–	$\varvec{p<0.001}$	$\varvec{p=0.009}$	$p=0.996$
Redox	MDM	Data density (EB)	−0.09%	0.42%	0.06%	−0.05%
			$p=0.986$	$\varvec{p<0.001}$	$p=0.055$	$p=0.900$
		MCDO	0.01%	0.18%	0.05%	0.01%
			$p=0.377$	$\varvec{p=0.001}$	$p=0.102$	$p=0.487$
		OOD only	–	2.09%	0.41%	−0.10%
			–	$\varvec{p<0.001}$	$\varvec{p<0.001}$	$p=0.977$
Redox	GBM	Data density (EB)	0.02%	0.21%	0.06%	0.01%
			$p=0.168$	$\varvec{p<0.001}$	$\varvec{p=0.003}$	$p=0.265$
		GBM	0.02%	0.12%	0.05%	0.02%
			$p=0.123$	$\varvec{p=0.007}$	$\varvec{p=0.013}$	$p=0.076$
		OOD only	-	1.60%	0.11%	−0.29%
			-	$\varvec{p<0.001}$	$\varvec{p=0.025}$	$p=0.994$

For the density method, EB refers to embedding-based similarity. Shown here are the percentage decrease in RMSE compared to random sampling. Shown below are the *p*-values of corresponding paired t-tests with the alternative hypothesis of AL performing better than random sampling. Significant test results are bold

Next, we aim to probe the performance of the uncertainty estimates for application to active learning. We focus on the MDM and GBM models and perform uncertainty-based AL sampling using MCDO and embedding-based density UQ methods for each model. Table 6 shows the mean percentage and the associated *p*-value of a paired t-test by which AL improves on random sampling for both predictive tasks, the selected modeling approaches, and the selected UQ approaches. These results are averaged across the different starting training data sizes and sample sizes. We show results for both the standard active learning setup where the query set is drawn from the same distribution as the training data and for the generalization-probing setup where a set of molecules is artificially removed from the initial training set as OOD molecules. For the second case, we observe whether active learning is beneficial relative to random sampling for improving performance on the OOD molecules, the in-distribution (ID) molecules, and the full test set consisting of both OOD and ID molecules. Additionally, we compare the UQ-driven AL approaches to a sampling approach which directly samples only molecules from the OOD bin. This is not possible in real applications as we are using our prior knowledge of which molecules were artificially removed. However, this provides a useful upper bound on the possible improvement rate for the OOD molecules.

The primary result is that under most conditions uncertainty-based active learning leads to improved performance compared to random sampling. Even though the magnitude of the improvement is generally small, the difference in performance is statistically significant for many instances, particularly for performance on OOD data. It is important to note that our study only experiments with a single iteration, and the quantity of molecules being incorporated is limited. Despite the small magnitude, such statistically significant improvements over multiple iterations could compound, potentially leading to substantial differences in performance over time. Overall, the results are similar across the different target properties (solubility and redox potential) and models (MDM and GBM). When performing active learning in a standard setup, AL shows mixed but somewhat beneficial performance relative to random sampling. For solubility prediction, AL provides the most benefit for the MDM model with both UQ approaches performing equally well. For redox potential prediction, the results are more mixed, with the GBM model and the embedding-based density method showing the strongest benefit.

When performing AL in the generalization-probing setup, the best performing UQ approaches show larger benefits than for applying AL in the standard setup. In particular, the embedding-based data density method consistently shows a larger improvement for the OOD molecules than is achieved in the standard setup. This shows that AL can provide a boost to the rate at which a model can generalize to previously unseen regions of molecular structure space. However, in this setup the improvement levels for the test set as a whole are smaller than OOD while performance on the ID molecules is not accelerated relative to random sampling. Our primary focus is on the performance with OOD data. Generally, AL exhibits superior results for both OOD and the whole test set. While there is some trade-off in ID performance, the compromise is not as drastic as in the OOD-only approach. Therefore, the balance achieved by AL, with significant improvement on OOD and whole test set data and reasonable performance on ID data, affirms its value in balancing between accelerated generalization and maintaining ID performance.

We also included additional baseline utilizing a model-free, purely diversity-based selection method for the solubility task using the MDM model. This technique essentially gives a higher likelihood of selection to molecules in the training set that are further apart from the rest. The similarity measurement here is based on the embedding approach. The results obtained using this diversity-based method are shown in Table 6. It is worth noting that this diversity-driven method did not yield significantly different results from random sampling when applied to the traditional active learning task. It did outperform random sampling in the context of OOD molecules. However, it underperformed in relation to both the entire test set and the ID set.

**Fig. 9:**
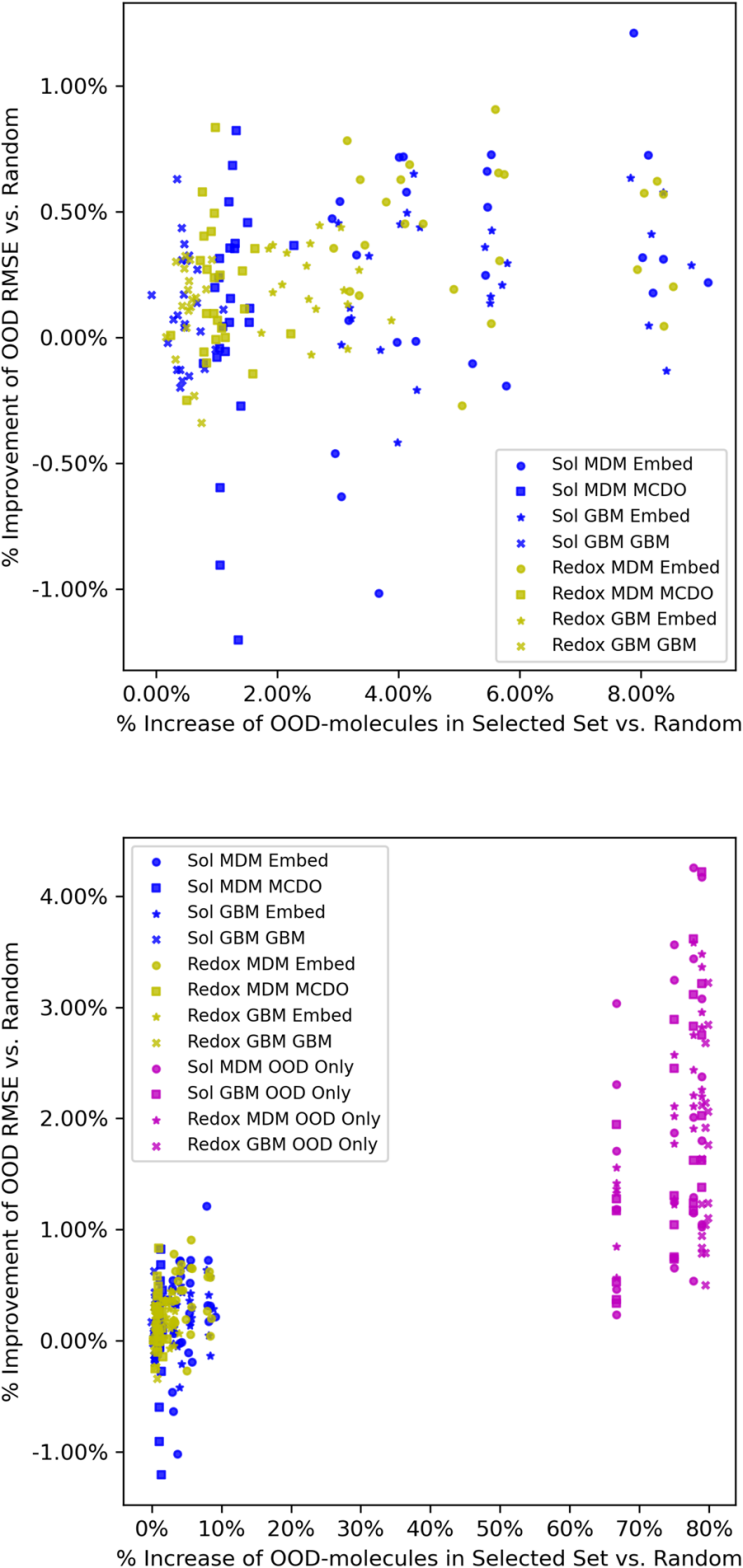
The impact of the proportion of OOD molecules sampled on the improvement in OOD RMSE relative to a random sampling strategy for the UQ-based AL (top) and with the OOD-only benchmark results included (bottom)

Since sampling only OOD molecules provides a significantly larger boost to performance, we might expect that AL methods which more successfully sample larger portions of OOD molecules will see a larger boost in performance. In Fig. 9, we explore the relationship between the percentage of AL selected molecules belonging to the missing bin and the resulting performance improvement relative to random sampling. We find that almost all AL sampling methods result in more OOD molecules being sampled than the random strategy. However, we find a very weak dependence of the resulting performance improvement on this factor. The significant variability in the observed improvement given the same number of sampled OOD molecules indicates that performance improvements are not purely driven by sampling molecules from the missing bin but by specifically which molecules are being sampled. Although the dependence is weak, we do observe a linear increasing trend when the results from the OOD-only sampling method are added to the plot (lower plot of Fig. 9). The vertical lines on the right side of the lower plot correspond to the four starting percentages of training data, and the OOD-only points largely align with these lines. However, the exact position of each point can deviate slightly because it is impacted by the degree of randomness inherent in the random sampling method because what is shown here is the difference between the OOD-only methods and random sampling.

**Fig. 10:**
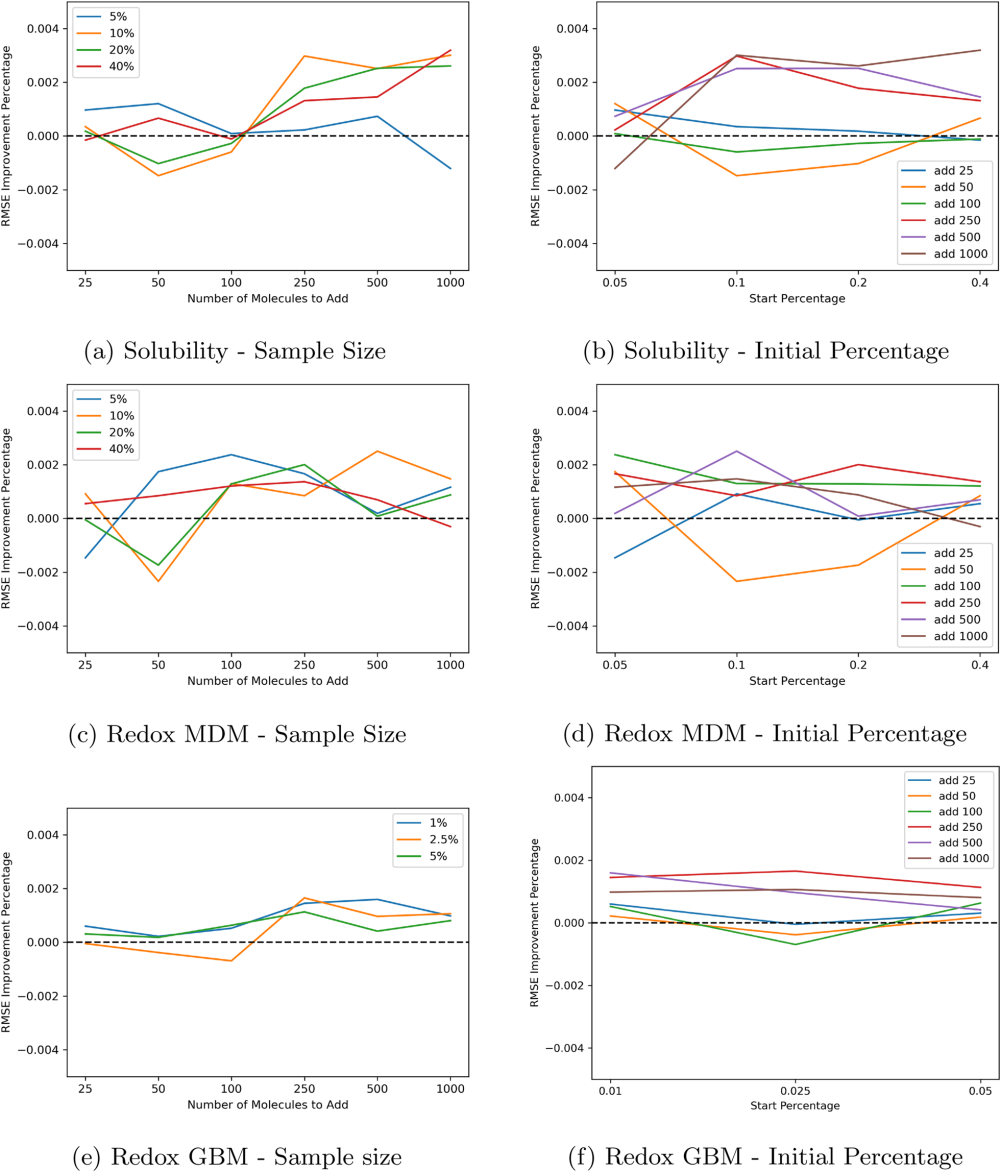
Dependence of active learning performance improvement on data size factors including the percentage of data used for the initial training step and the sample size of new datapoint selected using AL

We explore whether data set size properties affect the impact of AL on model performance. In Fig. 10, we show the mean AL performance improvement as a function of the starting percentages of training data or the number of molecules to add during active learning. Each data point is the average result of 30 active learning experimental runs. We find that performance tends to improve slightly for larger amounts of data being sampled during the AL step. It also appears that very low initial amounts of training data may inhibit the benefit of AL, possibly due to the inaccuracies of the UQ estimation with limited data availability. These trends are both clearer for the solubility prediction task than for the redox potential prediction task. These conclusions demonstrate that it would be a challenge to apply these methods in real practical applications where it is likely infeasible to collect data sets of the required sizes (e.g. AL samples of greater than 250 molecules).

Finally, we studied the relationship between the performance of the uncertainty estimation performance of each UQ method and its performance on the downstream AL task. The correlations between active learning results and UQ metrics are shown in Table 7. We find that UQ methods that perform well according to the OOD UQ metrics ($\rho _{\text {ood}}$ and $\rho _{\Delta \text {error}}$) also show improved performance on the AL generalization task while showing reduced performance on the ID AL task. In contrast UQ methods which are well-calibrated (ENCE) and show good error correlation ($\rho _{\text {error}}$) show stronger performance on the standard AL task and the ID AL task. This shows that inaccuracies in OOD uncertainty estimates likely limit the utility of uncertainty-driven AL sampling when the molecular library contains structurally dissimilar molecules from the original training set.

**Table 7:** Correlations of AL results with UQ metrics

	Generalization			Standard
Metric	OOD Bin	Whole	ID Bins	Whole
ENCE	−0.40	−0.32	0.40	0.40
$\rho _{\text {error}}$	−0.31	−0.29	0.20	0.07
$\rho _{\text {ood}}$	0.89	−0.01	−0.77	−0.14
$\rho _{\Delta \text {error}}$	0.69	0.23	−0.33	0.13

## Conclusions

In this study, we demonstrate significant limitations of current UQ and AL methods in application to practical molecular property prediction tasks relevant to material design for energy storage and other applications. We find that existing UQ methods fail to achieve strong performance across different evaluation dimensions meaning that individual UQ methods are specialized to specific use cases. However, we do find that UQ approaches perform consistently across different target properties and mostly consistently for different modeling architectures leading to generalizable conclusions about UQ performance. In particular, we find that most commonly used UQ methods perform poorly at evaluation metrics that probe performance on OOD molecules. Instead, simple nearest neighbor-based density estimates outperform the UQ techniques on this evaluation dimension. This UQ performance results translate into downstream AL performance, as data density-based methods show more effective selection of under-sampled molecule types to support accelerated model generalization.

Crucially, we demonstrate the AL performance strongly depends on whether the method is being applied to purely in-distribution data or is being applied to novel types of molecules that were not observed during training. The second is likely to be the case in many practical applications, where experimenters are seeking to fill in existing gaps in available training data to support broader applicability of property prediction models. Common evaluation techniques which rely on random samples of the currently available training data fail to capture much of the behavior of both UQ and AL methods in this scenario.

Our work has identified several key research gaps and future directions. The first is the development of UQ methods that are effective at estimating uncertainties for both in-domain and out-of-domain molecules and at providing both relative and calibrated information about expected errors. Additionally, we find that UQ-guided active learning provides statistically significant but magnitude-wise modest improvement in model performance relative to random sampling and might not be able to currently address the challenges of limited resource experimental efforts due to the dependence of AL success on having sufficiently sized data samples. Further work is needed to improve the learning ability of models in the low-data regime and accelerate the ability to generalize from small amounts of targeted data collection.

## Data Availability

The source code is available on GitHub at https://github.com/pnnl/UQALE. The aqeuous solubility dataset was published by [8] and accessible at https://doi.org/10.6084/m9.figshare.14552697. The redox potential dataset was published by [19] and accessible at https://github.com/piyushtagade/SLAMDUNCS.
